# Synthesis, Characterization, Electrochemical Studies, and *In Vitro* Antibacterial Activity of Novel Thiosemicarbazone and Its Cu(II), Ni(II), and Co(II) Complexes

**DOI:** 10.1155/2014/592375

**Published:** 2014-01-09

**Authors:** Salman A. Khan, Abdullah M. Asiri, Khalid Al-Amry, Maqsood Ahmad Malik

**Affiliations:** ^1^Chemistry Department, Faculty of Science, King Abdulaziz University, P.O. Box 80203, Jeddah 21589, Saudi Arabia; ^2^Center of Excellence for Advanced Materials Research (CEAMR), King Abdulaziz University, P.O. Box 80203, Jeddah 21589, Saudi Arabia

## Abstract

Metal complexes were prepared by the reaction of thiosemicarbazone with CuCl_2_, NiCl_2_, CoCl_2_, Cu(OAc)_2_, Ni(OAc)_2_, and Co(OAc)_2_. The thiosemicarbazone coordinates to metal through the thionic sulfur and the azomethine nitrogen. The thiosemicarbazone was obtained by the thiosemicarbazide with 3-acetyl-2,5-dimethylthiophene. The identities of these compounds were elucidated by IR, ^1^H, ^13^C-NMR, and GC-MS spectroscopic methods and elemental analyses. The antibacterial activity of these compounds was first tested *in vitro* by the disc diffusion assay against two Gram-positive and two Gram-negative bacteria, and then the minimum inhibitory concentration (MIC) was determined by using chloramphenicol as reference drug. The results showed that compound 1.1 is better inhibitor of both types of tested bacteria as compared to chloramphenicol.

## 1. **Introduction**

Most of the tropical and subtropical regions of the world are suffering from parasitic diseases. The intestinal causative agent of amoebiasis is protozoan parasite *Entamoeba histolytica*, which is affecting millions of people throughout the world. Millions of people die every year because of these diseases [1]. Major side effects such as neurological complications and the possible selection of a resistant *E. histolytica* strain have been reported by using metronidazole [2]. Therefore, it has become a topic of interest for the researchers to find some new amoebicidal drug and the treatment of infectious diseases still remains an important and challenging problem. Numerous compounds have been synthesized to treat such infectious diseases, but their medical use has been limited by their relatively high toxicity, bacterial resistance, and pharmacokinetic deficiencies. It is found that transition metals and their complexes have varying utility and interesting chemistry. Metal complexes with suitable ligands are said to be chemically more significant and specific than the metal ions [3]. Preparation of transition metal complexes with thiosemicarbazone ligands has been paid considerable attention due to the pharmacological properties of both ligands and complexes [4, 5]. Thiosemicarbazone derivatives form an important class of organic compounds due to their structural chemistry and biological activities, such as antibacterial, antivirus activities. Heterocyclic sulfur and oxygen containing thiosemicarbazones have been the subjects of extensive investigation because of their use for the biological applications [6, 7]. Thiosemicarbazones have received great interest because of their bonding modes, biological implications, structural diversity, and ion-sensing ability [8]. The biological application and properties of metal complexes differ from those of either ligands or the metal ions, and increased and/or decreased biological activities of transition metal complexes like Cu(II) are reported in the literature [9–11]. Thiosemicarbazone compounds are also applicable in fields of inorganic chemistry. They are used as a chelating ligand for the formation of metal complexes because of variety of flexible donor sets of sulfur and nitrogen. Cu(II), Ni(II), and Co(II) metal complexes of thiosemicarbazone dramatically increase the biological activities such as antibacterial, antifungal, anti HIV, and anti-inflammatory [12]. Thiosemicarbazones and their metal complexes are also applicable in the field of material sciences such as nonlinear optical (NLO) [13], electrochemical sensing [14], and Langmuir film [15]. Various thiosemicarbazone derivatives and their metal complexes are notable materials for their second harmonic generation (SHG) [16]. People are working from last many years on the synthesis and characterization of transition metal complexes with thiosemicarbazones [17–20], but very little work has been done so far on Cu(II), Ni(II), and Co(II) complexes with substituted thiosemicarbazone ligands. Metal thiosemicarbazone complexes are promising as new class of experimental anticancer chemotherapeutic agents which shows evidence of inhibitory behavior against cancer [21]. These are also a useful model for bioinorganic processes [22, 23]. Due to wide range of application of the thiosemicarbazones and their metal complexes, we have synthesized, characterized, and studied the antibacterial activity of thiosemicarbazone and its Cu(II), Ni(II), and Co(II) metal complexes.

## 2. **Experimental**

### 2.1. General

All melting points were measured with a capillary apparatus and are uncorrected. All the compounds were routinely checked by IR, ^1^H NMR, mass spectrometry, and elemental analysis. IR spectra were recorded in KBr discs on a Perkin-Elmer model 1620 FTIR spectrometer. ^1^H NMR spectra were recorded at ambient temperature using a Bruker spectrospin DPX-600 MHz spectrometer in CDCl_3_ and DMSO-d_6_. The following abbreviations were used to indicate the peak multiplicity as s: singlet, d: doublet, t: triplet, and m: multiplet. FAB mass spectra were recorded on a JEOL SX102 mass spectrometer using argon/xenon (6 kV, 10 mB gas). The reactions were monitored by precoated aluminium silica gel 60F 254 thin layer plates procured from Merck (Germany).

Electrochemical properties of the synthesised metal complexes were examined with CH Instruments, USA (Model 1110A-Electrochemical analyzer, Version 4.01), in CHCl_3_ in the presence of tetrabutylammonium hexafluorophosphate (0.10 mol L^−1^) as supporting electrolyte. All CV measurements were recorded at room temperature with a conventional three-electrode configuration consisting of a platinum wire working electrode, a platinum counter electrode, and a SCE (saturated calomel electrode) reference electrode under argon. Electrochemical band gaps were calculated from onset potentials of the anodic and cathodic waves. In order to improve the sensitivity and resolution of the voltammetric peaks and to provide a reproducible active surface, the glassy carbon electrode was polished to a mirror finish with 0.3-micron alumina on a smooth polishing cloth and then rinsed with methanol and double distilled water prior to each electrochemical measurement. All the metal complex solutions investigated by electrochemical techniques were purged for 10 min with water-saturated nitrogen.

### 2.2. Synthesis of Thiosemicarbazone: A General Method

**Scheme 1:**
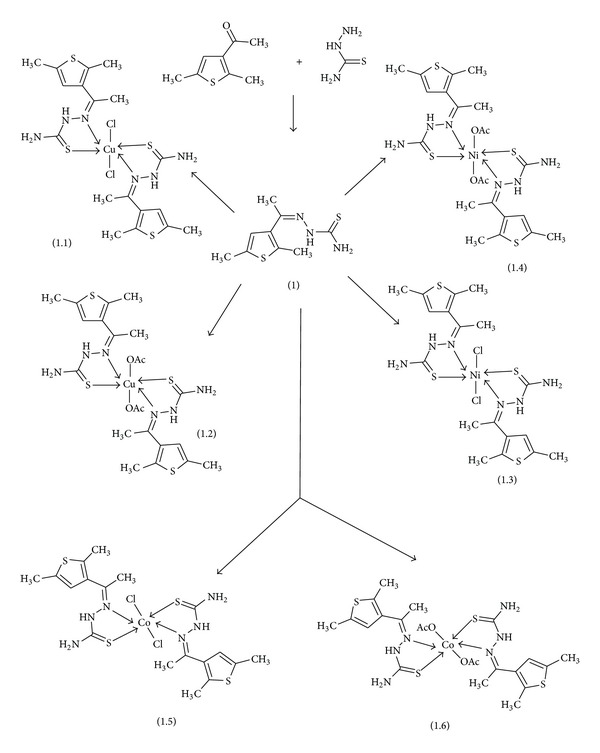


Thiosemicarbazone was synthesized (Scheme 1) by refluxing the solution of thiosemicarbazide (0.03 mol) in methanol and the alcoholic solution of 3-acetyl-2,5-dimethylthiophene (0.03 mol) at 60°C for 5 h with continuous stirring. After cooling the compounds were filtered and recrystallized from methanol [24, 25].

*(2E)-2-[1-(2,5-Dimethylthiophen-3-yl)ethylidene]hydrazinecarbothioamide (1)*. Yield: 88%; m.p. 158°C; Anal. Calc. for C_9_H_13_N_3_S: C, 47.55; H, 5.76; N, 18.48; S, 28.21. Found: C, 47.48; H, 5.74; N, 18.42; S, 28.15. IR (KBr discs) *v*_max_ cm^−1^: 3400 (N–H_2_), 3286 (N–H), 2910 (C–H), 1612 (C=C), 1585 (C=N), 1144 (C–N), 1091 (N–N), 945 (C=S); ^1^H NMR (DMSO-d_6_) *δ*_H_: 10.85 (s, 2H, NH_2_), 8.92 (s, 1H, –NH), 6.74 (s, 1H, C–H_aromatic_) 2.52 (s, 3H, –CH_3_), 2.40 (s, 3H, –CH_3_), 2.16 (s, 3H, –CH_3_), ^13^C NMR (DMSO-d_6_) (**δ**): 173.23 (C=S), 148.50 (C=N), 22.1, 15.92 (CH_3_), 15.90 (CH_3_), 15.12 (CH_3_). Mass spectra (M^+●^) at *m/z*: 228 (60).

### 2.3. Preparation of Cu(II) Complex

A solution of cupric chloride/copper acetate (2 mmol) dissolved in minimum quantity of methanol was added with stirring to the hot solution of ligand (4 mmol) in methanol (20 mL) and the reaction mixture was heated under reflux for 3 h. On keeping the solution to ambient temperature for overnight, the solid formed was separated by filtration, washed with hot water, followed by small quantity of methanol, and dried. Recrystallization was carried out from methanol [26–29].

*C*_*18*_ *H*_*26*_ *N*_*6*_ *S*_*2*_ *CuCl*_*2*_ *(1.1)*. Yield: 78%; m.p. 182.4°C; Anal. Calc. for (C_18_H_26_N_6_S_2_CuCl_2_: C, 42.06; H, 5.06; N, 16.35; Found: C, 41.8; H, 4.96; N, 16.31; IR (KBr discs) *v*_max_ cm^−1^: 3423 (N–H_2_), 3220 (N–H), 2953 (C–H), 1577 (C=N), 1199 (C–N), 1075 (N–N), 925 (C=S), 435 (Cu–N), 322 (Cu–S); ^1^H NMR (DMSO-d_6_) *δ*_H_: 10.82 (s, 2H, N–H_2_). 8.62 (s, 1H, –NH), 6.81 (s, 1H, CH_aromatic_) 2.59 (s, 3H, –CH_3_), 2.48 (s, 3H, –CH_3_), 2.38 (s, 3H,–CH_3_); ^13^C NMR (DMSO-d_6_) (**δ**): 178.59 (C=S), 150.84 (C=N), 18.05 (CH_3_), 15.83 (CH_3_), 14.89 (CH_3_). Mass spectra (M^+●^) at *m/z*: 515 (55).

*C*_*22*_ *H*_*32*_ *N*_*6*_ *S*_*2*_ *O*_*4*_ *Cu (1.2)*. Yield: 75%; m.p. 172°C; Anal. Calc. for C_22_H_32_N_6_S_2_O_4_Cu: C, 47.01 H, 5.69; N, 14.95. Found: C, 46.94; H, 5.62; N, 14.88. IR (KBr discs) *v*_max_ cm^−1^: 3418 (N–H_2_), 3215 (N–H), 2922 (C–H), 1565 (C=N), 1195 (C–N), 1080 (N–N), 921 (C=S), 432 (Cu–N), 318 (Cu–S); ^1^H NMR (DMSO-d_6_) *δ*_H_: 10.85 (s, 2H, N–H_2_). 8.62 (s, 1H, –NH), 6.72 (s, 1H, CH_aromatic_) 2.65 (s, 3H, –CH_3_), 2.48 (s, 3H, –CH_3_), 2.38 (s, 3H, –CH_3_); ^13^C NMR (DMSO-d_6_) (**δ**): 175.52 (C=S), 151.36 (C=N), 18.26 (CH_3_), 16.03 (CH_3_), 15.04 (CH_3_). Mass spectra (M^+●^) at *m/z*: 563 (70).

### 2.4. Preparation of Ni(II) Complex

A solution of nickel chloride/nickel acetate (2 mmol) dissolved in minimum quantity of methanol was added with stirring to the hot solution of ligand (4 mmol) in methanol (20 mL) and the reaction mixture was heated under reflux for 3 h. On keeping the solution to ambient temperature for overnight, the solid formed was separated by filtration, washed with hot water, followed by small quantity of methanol, and dried. Recrystallization was carried out from methanol.

*C*_*18*_ *H*_*26*_ *N*_*6*_ *S*_*2*_ *NiCl*_*2*_ *(1.3)*. Yield: 80%; m.p. 232°C; Anal. Calc. for (C_9_H_13_N_3_S)_2_NiCl_2_: C, 41.64; H, 5.01; N, 16.19; S, Found: C, 41.58; H, 4.96; N, 16.15. IR (KBr discs) *v*_max_ cm^−1^: 3412 (N–H_2_), 3284 (N–H), 2975 (C–H), 1575 (C=N), 1162 (C–N), 1099 (N–N), 922 (C=S), 432 (Cu–N), 318 (Cu–S); ^1^H NMR (DMSO-d_6_) *δ*_H_: 10.80 (s, 2H, N–H_2_). 8.47 (s, 1H, –NH), 6.45 (s, 1H, CH_aromatic_) 2.64 (s, 3H, –CH_3_), 2.51 (s, 3H, –CH_3_), 2.41 (s, 3H, –CH_3_); ^13^C NMR (DMSO-d_6_) (**δ**): 194.00 (C=S), 146.80 (C=N), 15.90 (CH_3_), 15.82 (CH_3_), 15.05 (CH_3_). Mass spectra (M^+●^) at *m/z*: 521 (62).

*C*_*22*_ *H*_*32*_ *N*_*6*_ *S*_*2*_ *O*_*4*_ *Ni (1.4)*. Yield: 65%; m.p. 242°C; Anal. Calc. for C_22_H_32_N_6_S_2_O_4_Cu: C, 46.58; H, 5.60; N, 14.82; Found: C, 46.52; H, 5.56; N, 14.75; IR (KBr discs) *v*_max_ cm^−1^: 3429 (N–H_2_), 3280 (N–H), 3144 (C–H), 2988 (C–H), 1578 (C=N), 1148 (C–N), 995 (N–N), 927 (C=S), 430 (Cu–N), 320 (Cu–S); ^1^H NMR (DMSO-d_6_) *δ*_H_: 10.83 (s, 2H, N–H_2_). 8.50 (s, 1H, –NH), 6.52 (s, 1H, CH_aromatic_) 2.66 (s, 3H, –CH_3_), 2.55 (s, 3H, –CH_3_), 2.42 (s, 3H, –CH_3_); ^13^C NMR (DMSO-d_6_) (**δ**): 192.56 (C=S), 145.35 (C=N), 15.88 (CH_3_), 15.75 (CH_3_), 15.02 (CH_3_). Mass spectra (M^+●^) at *m/z*: 568 (74).

### 2.5. Preparation of Co(II) Complex

A solution of cobalt chloride/cobalt acetate (2 mmol) dissolved in minimum quantity of methanol was added with stirring to the hot solution of thiosemicarbazone (4 mmol) in methanol (20 mL) and the reaction mixture was heated under reflux for 3 h. On keeping the solution to ambient temperature for overnight, the solid formed was separated by filtration, washed with hot water, followed by small quantity of methanol, and dried. Recrystallization was carried out from methanol.

*C*_*18*_ *H*_*26*_ *N*_*6*_ *S*_*2*_ *CoCl*_*2*_ *(1.5)*. Yield: 85%; m.p. 248°C; Anal. Calc. for C_18_H_26_N_6_S_2_CoCl_2_: C, 41.62; H, 5.01; N, 16.18. Found: C, 41.58; H, 4.96; N, 16.14. IR (KBr discs) *v*_max_ cm^−1^: 3433 (N–H_2_), 3235 (N–H), 2927 (C–H), 1566 (C=N), 1143 (C–N), 1088 (N–N), 923 (C=S), 435 (Cu–N), 326 (Cu–S); ^1^H NMR (DMSO-d_6_) *δ*_H_: 10.85 (s, 2H, N–H_2_). 8.59 (s, 1H, –NH), 6.42 (s, 1H, CH_aromatic_) 2.63 (s, 3H, –CH_3_), 2.52 (s, 3H, –CH_3_), 2.43 (s, 3H, –CH_3_); ^13^C NMR (DMSO-d_6_) (**δ**): 188.50 (C=S), 145.57 (C=N), 15.85 (CH_3_), 15.72 (CH_3_), 15.11 (CH_3_). Mass spectra (M^+●^) at *m/z*: 522 (75).

*C*_*22*_ *H*_*32*_ *N*_*6*_ *S*_*2*_ *O*_*4*_ *Co (1.6)*. Yield: 82%; m.p. 263°C; Anal. Calc. for C_22_H_32_N_6_S_2_O_4_Co: C, 46.56; H, 5.64; N, 14.81, Found: C, 46.52; H, 5.60; N, 14.76; IR (KBr discs) *v*_max_ cm^−1^: 3432 (N–H_2_), 3265 (N–H), 3147 (C–H), 2918 (C–H), 1536 (C=N), 1163 (C–N), 1082 (N–N), 918 (C=S), 425 (Cu–N), 322 (Cu–S); ^1^H NMR (DMSO-d_6_) *δ*_H_: 10.82 (s, 2H, N–H_2_). 8.63 (s, 1H, –NH), 6.77 (s, 1H, CH_aromatic_) 2.59 (s, 3H, –CH_3_), 2.48 (s, 3H, –CH_3_), 2.41 (s, 3H, –CH_3_); ^13^C NMR (DMSO-d_6_) (**δ**): 190.25 (C=S), 143.48 (C=N), 16.54 (CH_3_), 15.80 (CH_3_), 15.06 (CH_3_). Mass spectra (M^+●^) at *m/z*: 569 (84).

### 2.6. Organism Culture and *In Vitro* Screening

The antibacterial activity was assessed by the disc diffusion method with minor modifications. The antibacterial cells *S. aureus, S. pyogenes, S. typhimurium*, and *E. coli* were subcultured in BHI medium and incubated for 18 h at 37°C, and then the bacterial cells were suspended, according to the McFarland protocol in saline solution to produce a suspension of about 10^−5^ CFU/mL: 10 *μ*L of this suspension was mixed with 10 mL of sterile antibiotic agar at 40°C and poured onto an agar plate in a laminar flow cabinet. Five paper disks (6.0 mm diameter) were fixed onto nutrient agar plate. 1 mg of each test compound was dissolved in 100 *μ*L DMSO to prepare stock solution from stock solution different concentration 10, 20, 25, 50, and 100 *μ*g/*μ*L of each test compound were prepared. These compounds of different concentration were poured over disk plate onto it. Chloramphenicol (30 *μ*g/disk) was used as standard drug (positive control) and DMSO poured disc was used as negative control. The susceptibility of the bacteria to the test compounds was determined by the formation of an inhibitory zone after 18 h of incubation at 36°C.  Table 1 reports the inhibition zones (mm) of each compound and the controls. The minimum inhibitory concentration (MIC) was evaluated by the macrodilution test using standard inoculums of 10^−5^ CFL/mL. Serial dilutions of the test compounds, previously dissolved in dimethyl sulfoxide (DMSO), were prepared to final concentrations of 512, 256, 128, 64, 32, 16, 8, 4, 2, and 1 *μ*g/mL to each tube was added 100 *μ*L of a 24 h old inoculum. The MIC, defined as the lowest concentration of the test compound, which inhibits the visible growth after 18 h, was determined visually after incubation for 18 h, at 37°C, and the results are presented in Table 2. DMSO and chloramphenicol were used as negative and positive controls, respectively.

## 3. **Results and Discussion**

**Figure 1:**
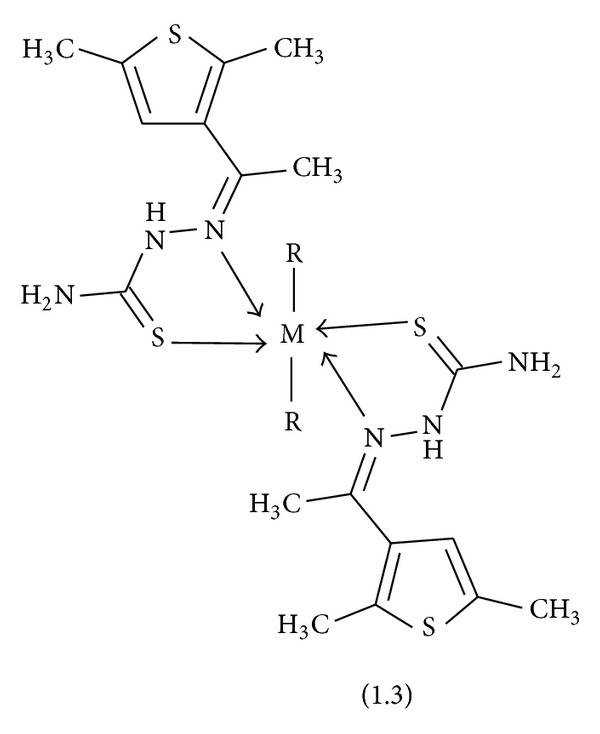
Structure of metal complexes, where R is AcO, Cl, and M is Cu(II), Ni(II), and Co(II).

Reaction of thiosemicarbazone with CuCl_2_, NiCl_2_, CoCl_2_, Cu(OAc)_2_, Ni(OAc)_2_, and Co(OAc)_2_ gave amorphous solid compounds. All the compounds are isolated in good yields and were stable both in the solid and solution states. Analytical data of these compounds are in good agreement with their composition (see Section 2). The complexes are insoluble in water, methanol, and ethanol and soluble in DMF and DMSO. The structures of the ligand and complexes are presented in Scheme 1 and Figure 1. The chemical structures of all the compounds were confirmed by means of elemental analyses and IR, ^1^H NMR, ^13^C NMR, and mass spectra analyses.

### 3.1. IR Spectral Studies

Assignments of selected characteristic IR band positions provide significant indication for the formation of thiosemicarbazone and its complexes. The thiosemicarbazone can exist in thione and thiol tautomeric forms shown in Figures 3(a) and 3(b), respectively.

However, the existence of a strong band in the region 945 cm^−1^ due to *v *(C=S) stretch, absence of any band in the region 2528–2675 cm^−1^ due to* v* (C–SH) stretch, and the presence of *v* (N–H) stretch at 3286 cm^−1^ in the spectra of the ligand suggest that the thiosemicarbazones remain in their thione form in the solid state.

The downward shift of *v* (C=S) band by (18–27) cm^−1^ in the complexes suggested the coordination of sulfur in thionic form. A strong band at 1585 cm^−1^ was assigned to *v* (C=N) stretch of azomethine linkage in the spectra of free thiosemicarbazone. In the complexes this band shifted to lower frequency by 7–49 cm^−1^ and this lowering was attributed to the coordination of azomethine nitrogen with metal and formation of M–N band.

### 3.2. NMR Spectral Analysis

Further evidence for the formation of thiosemicarbazone and its metal complexes was obtained from the ^1^H NMR, which provides diagnostic tools for the positional elucidation of the protons. Assignments of the signals are based on the chemical shifts and intensity patterns. The thiosemicarbazone does not show any resonance at ca. 4.0 ppm, attributed to –SH proton resonance, while the appearance of a broad peak at 8.56 ppm due to the –NH proton of thioamide group indicates that even in a polar solvent such as DMSO-d_6_ they remain in the thione form. The –NH proton signal of the thiosemicarbazones usually shifts 0.20–0.45 ppm upfield in their respective complexes.

The ^13^C NMR spectrum of the thiosemicarbazone was recorded in DMSO-d_6_ and the spectral signals are in good agreement with the probable structures. The thiosemicarbazone showed two signals at 175.52 and 151.36 ppm assigned due to thioamide (C=S) and azomethine carbon (C=N), respectively. ^13^C NMR spectra also provide diagnostic tools for the elucidation of the coordination mode of the thiosemicarbazone in complexes. Assignments of the signals are based on the chemical shifts and intensity patterns and coordination induced shift (CIS), Δ*δ* [Δ*δ* = *δ* (complex) −  *δ* (free ligand)] the signals for carbon atom in the vicinity of the coordinating functions. Thus the C=S carbon in thiosemicarbazone experiences CIS value of 2.29–20.77 ppm in complexes indicating the coordination of thione sulphur. As a result of variation of electron density on coordination, azomethine carbon signal is shifted downfield by 0.2–20.77 ppm in their respective complexes, which indicates coordination of nitrogen lone pair to metal. Other carbons in these complexes resonate nearly at the same frequency as that of thiosemicarbazone as given in the experimental.

### 3.3. FAB Mass Analysis

Characteristic peaks were observed in the mass spectra of thiosemicarbazone and its metal complexes, which followed the similar fragmentation patterns. The spectrum of thiosemicarbazone shows a molecular ion peak (M^+●^) at *m/z* = 228 and its complexes do the following: Cu(II) (1.1 and 1.2) shows a molecular ion peak (M^+●^) at *m/z* = 515 and 563, Ni(II) (1.3 and 1.4) shows a molecular ion peak (M^+●^) at *m/z* = 521–568, and Co(II) (1.5 and 1.6) shows a molecular ion peak (M^+●^) at *m/z* = 522 and 569.

### 3.4. Antimicrobial Activity: Disc Diffusion

The *in vitro *antibacterial activity of thiosemicarbazone and its metal complexes (1–1.6) was assayed by the disk diffusion method using cultures of *S. aureus, S. pyogenes, S. typhimurium*, and *E. coli*. Chloramphenicol (30 mg) was used as the standard drug, whereas a DMSO-wetted disk was used as negative control [25]. The results showed that compounds 2 and 3 are better at inhibiting the growth of both types of the bacteria (Gram-positive and Gram-negative) as compared to chloramphenicol. Results are summarized in Tables 1 and 2.

### 3.5. Electrochemical Studies

**Figure 2:**
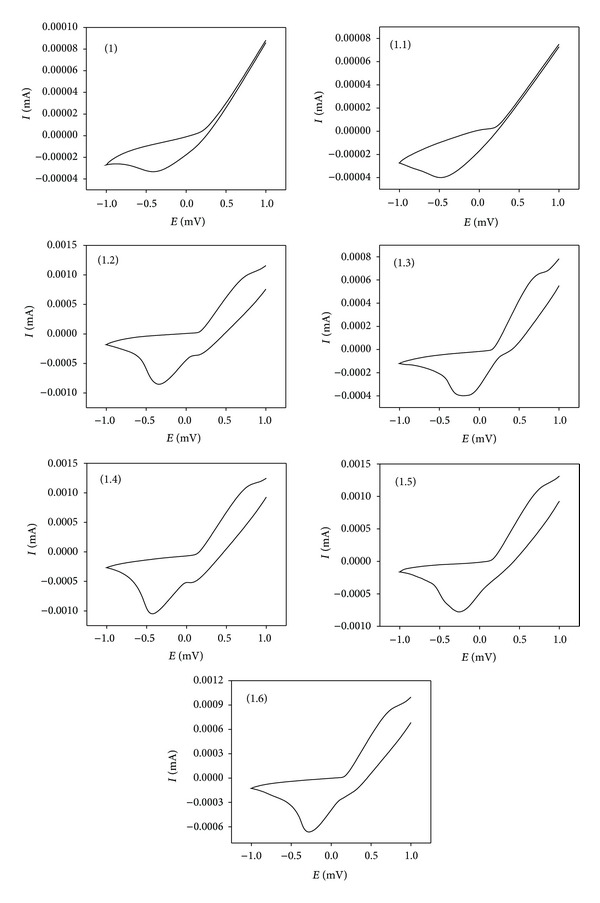
Cyclic voltammogram of ligand and its metal complexes.

**Figure 3:**
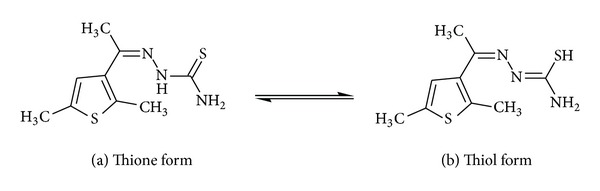


Generally the electrochemical properties of the complexes depend on a number of factors such as chelate ring size, axial ligation degree, distribution of unsaturation, substitution pattern in the chelate ring, charge type, and coordination number [30, 31]. The redox behaviors of thiosemicarbazone and its complexes have been investigated by cyclic voltammetry in the range from +1.6 to −2.0 V. The cyclic voltammograms of the complexes show metal centered processes and waves corresponding to the ligand. Repeated scans, as well as different scan rates, showed that dissociation does not take place in these complexes. The electrochemical data of the complexes are given in Table 3 and the representative cyclic voltammograms for the behaviours of the complexes are shown in Figure 2. The cyclic voltammogram of the ligand 1 in DMF shows an irreversible anodic waves in the positive range (0.989 V) and irreversible cathodic wave in the negative range (−0.50 V), which can be attributed to the redox behaviors of the imine or thioamide groups present in the ligand [32]. In metal chlorides (1.1, 1.3, and 1.5), addition to the peaks of ligand, exhibits a single quasireversible anodic wave and a single irreversible cathodic wave. In the positive range 0 to +1.00 V, a redox couple associated with the M(III)/M(II) process can be observed. The peak of the M(III)/M(II) couple is similar to the values reported earlier [33]. The redox process is quasireversible in nature as is evident from the criteria: the *E*_1/2_ values are independent of scan rate; the Δ*E*_*p*_ slightly increases with increasing scan rate (50–600 mV·s^−1^) and is always greater than 60 mV. Cyclic voltammogram of acetate complex is shown in Figure 2 (1.2, 1.4, and 1.6). The complex exhibited two quasireversible peaks. Cyclic voltammogram displays two reduction peaks with an associated oxidation peak in the range of 0.20–0.85 V and 0.25–0.59 V. However, the peak current increases with the increase of the square root of the scan rates. This establishes the electrode process as diffusion controlled.

## 4. Conclusion

This research examined the synthesis, characterization, and antibacterial activity of novel thiosemicarbazone and its Cu(II), Ni(II), and Co(II) complexes. The thiosemicarbazone were obtained by the thiosemicarbazide with 3-acetyl-2,5-dimethylthiophene. *In vitro* antibacterial activity of these compounds was tested by the disk diffusion microdilution assay against two Gram-positive and Gram-negative bacteria. The results showed that chloro containing Cu(II) metal complex is better antibacterial agent as compare to chloramphenicol.

## Figures and Tables

**Table 1:** Antibacterial activity of thiosemicarbazone and its metal complexes, positive control chloramphenicol (Chlora.) and negative control (DMSO) measured by the halo zone test (unit, mm).

Compounds	Corresponding effect on microorganisms			
	*S. aureus *	*S. pyogenes *	*S. typhimurium *	*E. coli *
1	9.2 ± 0.3	9.0 ± 0.2	8.8 ± 0.2	8.2 ± 0.5
1.1	17.2 ± 0.2	17.6 ± 0.3	16.8 ± 0.4	20.2 ± 0.4
1.2	15.2 ± 0.4	15.8 ± 0.3	15.4 ± 0.3	16.8 ± 0.8
1.3	11.6 ± 0.2	11.2 ± 0.3	12.6 ± 0.4	11.8 ± 0.4
1.4	12.4 ± 0.4	11.8 ± 0.2	12.2 ± 0.4	11.6 ± 0.5
1.5	11.6 ± 0.3	12.4 ± 0.4	13.2 ± 0.4	12.6 ± 0.4
1.6	11.2 ± 0.4	12.2 ± 0.4	12.4 ± 0.5	13.2 ± 0.2
Chloro.	17.0 ± 0.5	18.2 ± 0.4	17.2 ± 0.8	20.0 ± 0.2
DMSO	—	—	—	—

**Table 2:** Minimum inhibition concentration (MIC) of thiosemicarbazone and its metal complexes.

Bacterial strain	MIC (*μ*gmL^−1^) compound							Positive control
	1	1.1	1.2	1.3	1.4	1.5	1.6	
*S. aureus *	512	32	64	128	64	128	64	32
*S. pyogenes *	512	32	64	128	128	256	128	32
*S. typhimurium *	128	32	32	256	128	256	128	32
*E. coli *	128	32	32	256	256	128	64	32

**Table 3:** Electrochemical properties of the compounds 1–1.6.

Compounds	Epa	Epc	ΔEp
1	0.989	0.50	
1.1	0.40	−0.60	0.39
1.2	0.26, 0.65	0.32, −0.52	0.45
1.3	0.32, 0.73	0.41, −0.39	0.40
1.4	0.29, 0.84	0.34, −0.59	0.78
1.5	0.33, 0.69	−0.36	0.61
1.6	0.32, 0.79	0.29, −0.43	0.65
